# Resolved lower limb muscle tone abnormalities in children with HIV encephalopathy receiving standard antiretroviral therapy

**DOI:** 10.1186/s12981-015-0085-4

**Published:** 2015-12-30

**Authors:** Theresa N. Mann, Kirsten A. Donald, Kathleen G. Walker, Nelleke G. Langerak

**Affiliations:** Division of Neurosurgery, Department of Surgery, H53 Old Main Building, Groote Schuur Hospital, University of Cape Town, Observatory 7925, Cape Town, South Africa; Division of Orthopaedic Surgery, Department of Surgical Sciences, Stellenbosch University, Tygerberg, Cape Town, South Africa; Division of Developmental Pediatrics, Department of Pediatrics and Child Health, University of Cape Town, Cape Town, South Africa; Division of Pediatric Neurology, Department of Pediatrics and Child Health, University of Cape Town, Cape Town, South Africa; Red Cross War Memorial Children’s Hospital, Cape Town, South Africa

**Keywords:** Spastic diplegia, HIV encephalopathy, Pediatric HIV

## Abstract

**Background:**

This short report arose from a follow-up study of children previously diagnosed with human immunodeficiency virus (HIV) encephalopathy and spastic diplegia and is among the first to describe that increased lower limb muscle tone in children with a confirmed HIV encephalopathy diagnosis may resolve over time in some cases.

**Results:**

Of 19 children previously diagnosed with HIV encephalopathy and increased lower limb muscle tone, some were found to have resolved muscle tone abnormalities during a follow-up physical examination [resolved group, *n* = 13, median age 9 years 7 months (interquartile range 7 years 3 months–10 years 9 months)] whereas others continued to show increased lower limb muscle tone at follow-up [unresolved group, *n* = 6 median age 8 years 6 months (interquartile range 7 years 9 months–9 years 7 months)]. A review of clinical records showed no significant differences in age or follow-up time between the resolved and unresolved groups. However, the unresolved group appeared to have severe disease at an earlier age than the resolved group, based on the age at antiretroviral treatment initiation [median age at start of treatment 2 years 3 months (interquartile range 7 months–5 years 3 months) vs. 8 months (interquartile range 6–12 months), *p* = 0.08] and had more severe neurological signs at the initial assessment.

**Conclusions:**

It is anticipated that this information may be of immediate value to those involved in the treatment of children with HIV encephalopathy and increased lower limb muscle tone whilst awaiting the outcome of future controlled clinical trials.

## Background

It is estimated that at least 210,000 children in South Africa are infected with human immunodeficiency virus (HIV) with vertical transmission identified as the most common mode of pediatric HIV infection [1]. HIV may penetrate the central nervous system (CNS) early in infection and children infected with HIV during the perinatal period may subsequently develop HIV encephalopathy (HIVE) [2, 3]. For example, it was recently reported that of 145 children seen at an HIV Neurology clinic at the Red Cross War Memorial Children’s Hospital in Cape Town between 2008 and 2012, 87 children (60 %) could be regarded as having an isolated diagnosis of HIVE [4]. Furthermore, 55 (63 %) of the children were reported as having spastic diplegia, including pathological gait patterns, as part of the HIVE diagnosis [4].

Little is known about the natural history of spastic diplegia in children with HIVE and with this in mind, a study investigating this topic was planned and approved by the University of Cape Town Human Research Ethics Committee (HREC 447/2012). Participants for the study were recruited from the afore-mentioned HIV Neurology clinic, which has ethical approval to collect information on a password protected database (HREC 126/201). It was anticipated that the original neurological findings would be confirmed during the screening of potential participants. However, more than two-third of the children screened no longer showed any evidence of spastic diplegia or increased muscle tone in the lower limbs.

Improvement in lower limb tone abnormalities in children with HIVE has been previously alluded to by Chiriboga et al. [5]. However, these observations fell beyond the main scope of the study and it remains unclear why increased muscle tone in the lower limbs of children diagnosed with HIVE appears to resolve in some children but not in others. In a similar way, this topic was not the aim of our planned research study. Nevertheless, we felt it important to report our clinical observations and offer preliminary insights into the possible role of (1) the current age of the children and follow-up time; (2) the severity of the initial neurological findings and; (3) age at the start of antiretroviral therapy (ART) on the subsequent resolution of increased lower limb muscle tone. It is anticipated that this information may be of immediate value to those involved in the treatment of children with HIVE and increased lower limb muscle tone whilst awaiting the outcome of future controlled clinical trials.

## Methods

### Participants

The current clinical observations occurred whilst screening possible participants for a study on the natural history of HIVE and spastic diplegia. Children potentially eligible for the study were identified from a database, which was compiled by two pediatric neurology specialists (KD and KW) at the HIV Neurology clinic of Red Cross War Memorial Children’s Hospital between 2008 and 2014. The database included each child’s medical history, the findings of a physical examination (PE 1) and the neurologist’s diagnostic conclusions.

Parents or caregivers were invited to bring their child to the hospital for counselling regarding informed consent and screening if the child appeared to meet the criteria for the study. All children had HIVE, diagnosed according to Centers for Disease Control (CDC) criteria [7]; were recorded as having spastic diplegia and/or increased muscle tone in the lower limbs; were ambulant; had no history of prematurity; no neurosurgical or orthopedic interventions; no botulinum toxin injections within the last 6 months; and currently were between 5 and 12 years of age.

### Outcome measures

During the screening, a highly experienced physiotherapist (NL) conducted a follow-up physical examination of the child’s lower limbs (PE 2). The assessment included testing muscle tone in the child’s hip flexor, rectus femoris, hamstring, adductor and plantar flexor muscles and visual inspection of the child’s walking and running gait pattern for abnormalities related to muscle spasticity. In addition, clinical records were used to gather information regarding each child’s (1) age and follow-up time; (2) severity of the initial neurological findings; and (3) age at the start of ART. Most recent CD4 and viral load (VL) results, dating from within approximately 1 year of PE 2, were also recorded along with the current ART regimen. The CNS penetration-effectiveness (CPE) score for each antiretroviral drug was taken from the revised CPE ranking of Letendre et al. [6] with higher scores indicating better effectiveness. Total CPE score for the regimen was taken as the sum of the CPE scores for each component.

### Data analysis

Children were divided into those who had normal lower limb muscle tone at PE 2 (resolved group) and those who continued to show increased lower limb muscle tone at PE 2 (unresolved group). All data was analyzed using Graphpad Prism (Version 6, Graphpad Software Inc, California, USA) and presented as the median value and inter-quartile range (IQR). A two-tailed Mann–Whitney *U* test was used to compare outcome measures in the resolved and unresolved groups with significance accepted at *p* < 0.05.

## Results

### Participants

Nineteen children identified from the database as potentially eligible for the study attended a screening session. Thirteen of the 19 children previously recorded with spastic diplegia or increased muscle tone in the lower limbs no longer showed any evidence of these signs (resolved group—6 boys, 7 girls) whereas the remaining six children continued to show increased muscle tone in the lower limbs (unresolved group—5 boys, 1 girl).

### Age and follow-up time

Table 1 provides an overview of age and follow-up time for the resolved and unresolved groups, respectively. There were no significant differences between the resolved and unresolved groups with respect to age at PE 1, PE 2 or follow-up time between PE 1 and PE 2.

**Table 1:** Overview of participants’ age at initial and follow-up physical examination

	Resolved group (*n* = 13)	Unresolved group (*n* = 6)	Resolved vs. unresolved
	Median (IQR)	Median (IQR)	*p* value
Age at PE 1 (years:months)	7:7 (4:0–9:1)	5:9 (3:9–6:9)	*p* = 0.19
Age at PE 2 (years:months)	9:7 (7:3–10:9)	8:6 (7:9–9:7)	*p* = 0.68
Time between PE 1 and PE 2 (years:months)	2:1 (1:2–3:4)	3:7 (1:3–5:3)	*p* = 0.23

*PE 1* initial physical examination, *PE 2* follow-up physical examination, *IQR* interquartile range

### Severity of the initial neurological findings

While all 19 children had shown increased lower limb muscle tone at PE 1, this finding had been recorded as “increased lower limb muscle tone” in some cases and “spastic diplegia” in other cases, indicating variation in the relative severity of the initial neurological findings. All 13 children in the resolved group were described as having “increased muscle tone” at PE 1, with none described as having “spastic diplegia”. In contrast, five of the six children in the unresolved group were described as having “spastic diplegia” at PE 1, with the remaining child described as having “increased muscle tone”.

### ART initiation, recent blood test results and current ART regimen

**Fig. 1:**
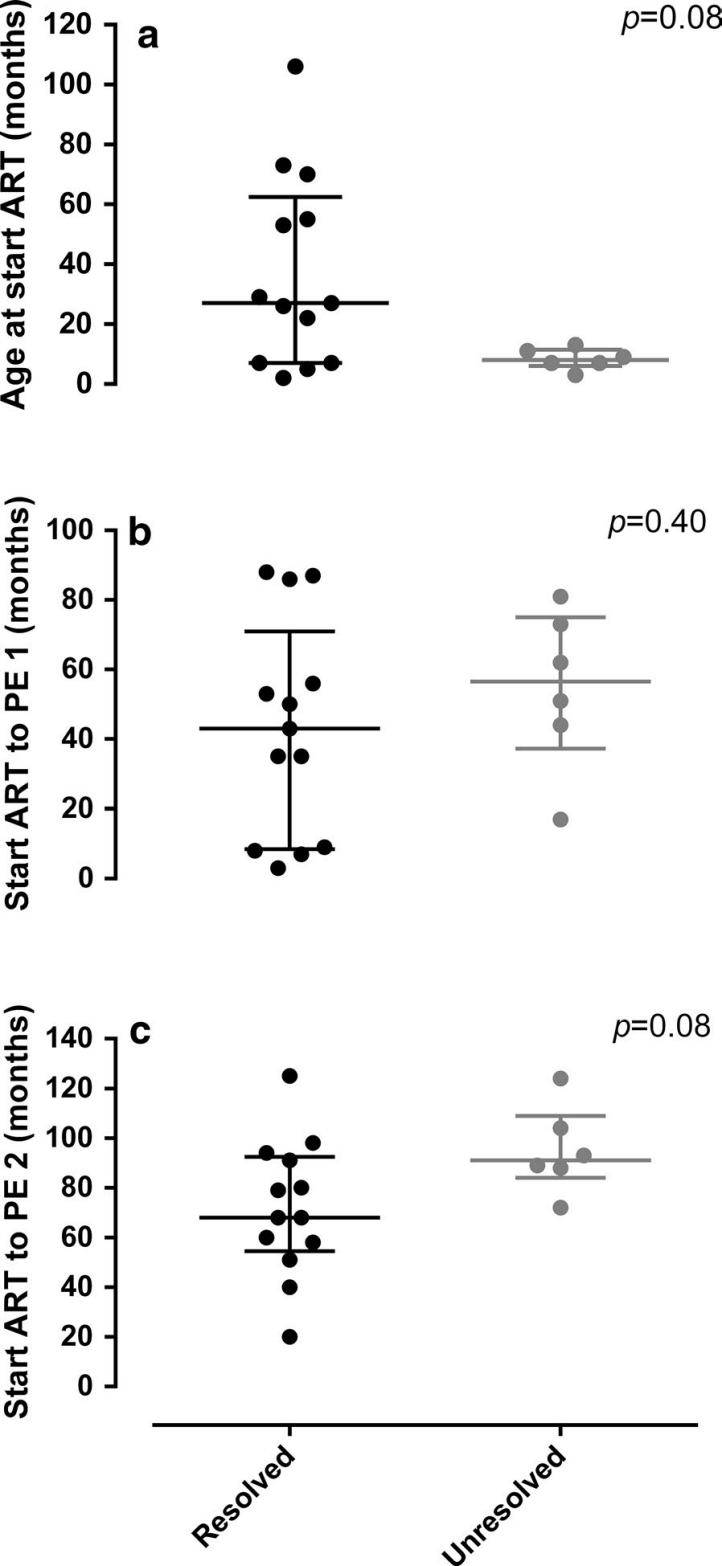
Difference between the resolved- and unresolved groups for: **a** age at the start of anti-retroviral therapy (ART); **b** time period from the start of ART to physical examination (PE) 1; and **c** time period from the start of ART to PE 2. Median value and inter-quartile range are indicated

There were no significant differences in the age at ART initiation, time on ART prior to PE 1 and time on ART prior to PE 2 (Fig. 1a–c). Most recent blood test results showed no immune suppression (*n* = 10) or moderate immune suppression (*n* = 2) within the resolved group according to CDC immunologic classification [7] with one result unavailable. All children in the unresolved group had no immune suppression. Most children in the resolved group had undetectable VL’s (*n* = 11) although two children had VL’s of log 4.2 and log 2.8, respectively. One child in the unresolved group had a VL of log 2.4 while the remaining five children had undetectable VL’s. Details of the current ART regimen were available for 11 of the children in the resolved group and all six of the children in the unresolved group. There were no noticeable distinctions in ART regimens or typical CPE scores between the groups, as shown in Table 2.

**Table 2:** Current antiretroviral regimens

*n*	NRTI	NRTI	NNRTI	PI	CPE score
Resolved group					
1	AZT	3TC		LPV/r	9
5	ABC	3TC		LPV/r	8
3	ABC	3TC	EFV		8
1	d4t	3TC		LPV/r	7
1	d4t	3TC	EFV		7
Unresolved group					
1	AZT	3TC	EFV		9
2	ABC	3TC		LPV/r	8
2	d4t	3TC		LPV/r	7
1	d4t	3TC	EFV		7

*NRTI* nucleoside reverse transcriptase inhibitor, *NNRTI* non-nucleoside reverse transcriptase inhibitor, *PI* protease inhibitor, *CPE* CNS penetration effectiveness, *AZT* zidovudine, *3TC* lamivudine, *EFV* efavirenz, *ABC* abacavir, *LPV/r* lopinavir/ritonavir, *d4t* stavudine

## Discussion

To our knowledge, this is one of the first reports to specifically describe that increased lower limb muscle tone in children with HIVE may resolve over time in some cases. This observation naturally led us to consider what factors might explain the difference between the resolved and unresolved groups and we posed a number of questions as follows.

### Is the resolution of increased lower limb muscle tone related to age?

There were no significant differences when comparing age at PE 1, age at PE 2 or the time period between PE 1 and PE 2 in the resolved and unresolved groups (Table 1). This would suggest that the current observations cannot necessarily be attributed to maturational factors.

### Is the resolution of increased lower limb muscle tone related to the severity of the initial neurological findings?

Those in the resolved group showed less prominent increases in muscle tone at PE 1 compared to those in the unresolved group. Unfortunately, there was no specific measure of muscle spasticity with which to firmly establish and quantify these differences. Nevertheless, the distinction between the groups would suggest that the resolution of increased lower limb muscle tone may indeed be associated with the severity of the initial neurological findings as distinguished by “spastic diplegia” or “increased lower limb muscle tone” at PE 1.

### Is the resolution of increased lower limb muscle tone related to the duration of ART?

Differences between the resolved and unresolved groups did not appear to be related to overall duration of ART with no significant differences in the duration on ART prior to PE 1 and PE 2 (Fig. 1b, c). Although there was a tendency towards a significant difference in duration on ART prior to PE 2 between the groups (Fig. 1c, *p* = 0.08), this tendency was towards shorter ART duration in the resolved group rather than the unresolved group and can be attributed to later ART initiation in the resolved group.

### Is the resolution of increased lower limb muscle tone related to age at the start of ART?

Although there was no significant difference in the age of ART initiation between the resolved and unresolved groups, all six children in the unresolved group had commenced ART by 13 months of age whereas only 4 of the 13 children in the resolved group had commenced ART within this time period.

Unfortunately, start of ART blood test results were not available and we were not able to ascertain the health status of the children when ART commenced. However, all children in the current study would have initiated ART under either the 2004 or 2010 South African National ART Guidelines, both of which required children to meet certain clinical criteria to be eligible for ART [8, 9]. Under the 2004 guidelines, infants and children were eligible for ART on the basis of (1) recurrent hospitalizations, (2) a modified World Health Organization Stage II or III disease or (3) a CD4 % <20 % [8]. In contrast, the 2010 guidelines allowed for ART initiation in all HIV-infected infants irrespective of health status but retained criteria of a clinical stage 3 or 4 or CD4 % ≤25 % in order to initiate ART in children 1–5 years of age [9].

None of the children in the current study initiated ART as an infant under the 2010 guidelines, therefore it is likely that all the children commenced ART due to a deterioration in health. It follows that all six children in the unresolved group may have had significant HIV disease progression within 13 months of age whereas only four of the children in the resolved group appear to have met one of the clinical criteria for ART initiation during infancy. The remaining nine children in the resolved group appear to have only developed significant disease progression somewhat later, at 22–106 months of age.

Initiation of ART within the first months of life may protect against HIV disease progression and improve short-term neurodevelopmental outcomes in HIV-infected infants who are not yet immune suppressed [10, 11]. However, it would appear that the association between early ART initiation and improved outcomes might not apply to infants whose health has already deteriorated prior to ART initiation. In other words, in the current study, those who initiated ART in infancy had poorer neurological outcomes than those who initiated ART somewhat later, most likely due to the former group having severe disease progression at an earlier age.

The current observations have a number of limiting factors including the small sample size and the absence of some key measurements with which to compare the resolved and unresolved groups. Missing data include immunological and viral load data from the start of ART and from PE 1, a means of quantifying muscle spasticity at PE 1 and PE 2 and more detailed information about ART regimens and longitudinal viral suppression. These limitations can be attributed to the fact that the current report was based on an unanticipated clinical observations rather than a planned research study. Nevertheless, this preliminary discussion could be of immediate relevance to healthcare professionals who may wish to consider the current findings when assessing the prognosis and orthopedic treatment options for children with HIVE who present with increased muscle tone in the lower limbs.

## Abbreviations

CDC: Centers for Disease Control
CNS: central nervous system
CPE: central nervous system penetration-effectiveness
HIV: human immunodeficiency virus
HIVE: HIV encephalopathy
ART: antiretroviral therapy
PE: physical examination
